# ALKBH5 promotes PD-L1-mediated immune escape through m6A modification of ZDHHC3 in glioma

**DOI:** 10.1038/s41420-022-01286-w

**Published:** 2022-12-24

**Authors:** Wenhui Tang, Ningbo Xu, Jian Zhou, Zhenyan He, Cameron Lenahan, Chenyang Wang, Huangyi Ji, Boyang Liu, Yujiao Zou, Huijun Zeng, Hongbo Guo

**Affiliations:** 1 Department of Neurosurgery Center, The National Key Clinical Specialty, The Engineering Technology Research Center of Education Ministry of China on Diagnosis and Treatment of Cerebrovascular Disease, Guangdong Provincial Key Laboratory on Brain Function Repair and Regeneration, The Neurosurgery Institute of Guangdong Province, Zhujiang Hospital, Southern Medical University, Guangzhou, 510282 China; 2 Department of Interventional Therapy, Zhujiang Hospital, Southern Medical University, Guangzhou, 510282 China; 3 Department of Neurosurgery, The Affiliated Tumor Hospital of Zhengzhou University, Zhengzhou, 450003 China; 4 Department of Biomedical Sciences, Burrell College of Osteopathic Medicine, Las Cruces, 88003 NM USA; 5 Department of Neurosurgery, The First Affiliated Hospital of Shantou University Medical College, Shantou, 515041 China; 6 Department of Radiation Oncology, Zhujiang Hospital, Southern Medical University, Guangzhou, 510282 China

**Keywords:** Immune evasion, Cancer therapy

## Abstract

N6-methylation of adenosine (m6A) is one of the most frequent chemical modifications in eukaryotic RNAs and plays a vital role in tumorigenesis and progression. Recently, emerging studies have shown that m6A modification by ALKBH5 was associated with immunotherapy response in various types of cancer. However, whether m6A demethylases ALKBH5 participate in regulating the tumor immune microenvironment and the efficacy of immunotherapy in glioblastoma remain unknown. Here, we found that deletion of ALKBH5 significantly inhibited the growth of glioma allografts, rescued the antitumoral immune response, and increased cytotoxic lymphocyte infiltration and proinflammatory cytokines in CSF while significantly suppressing PD-L1 protein expression. m6A-methylated RNA immunoprecipitation sequencing and RNA sequencing identify ZDDHC3 as the direct target of ALKBH5. Mechanically, ALKBH5 deficiency impairs the YTHDF2-mediated stability of ZDHHC3 mRNA, thereby suppressing PD-L1 expression by accelerating PD-L1 degradation in glioma. In addition, genetic deletion or pharmacological inhibition of ALKBH5 with IOX1 enhances the therapeutic efficacy of anti-PD-1 treatment in preclinical mice models. These data suggest that the combination of anti-PD-1 therapy and ALKBH5 inhibition may be a promising treatment strategy in glioma.

## Introduction

Gliomas are the most common primary and life-threatening intracranial tumors. Nearly half of glioma cases are diagnosed as glioblastoma (GBM), considered the most aggressive and lethal brain tumor with rapid progression, inevitable recurrence, and poor abysmal prognosis [1–3]. Although the strategy of surgical resection followed by chemotherapy and radiotherapy was used, the median overall survival for GBM patients is currently estimated to range from 12 to 16 months. Thus, novel treatment methods based on the biological attributes of GBM are urgently needed.

Immune checkpoint inhibitors (ICIs) have exhibited remarkable therapeutic success in numerous tumors, placing tumor immunotherapy in the spotlight [4, 5]. Among an array of available ICI strategies, blockade of programmed cell death 1 (PD-1)/programmed cell death ligand 1 (PD-L1) axis has achieved the most promising results in a variety of malignant tumors, including melanoma [6, 7], non-small cell lung cancer [8], renal cell carcinoma [9], and Hodgkin’s lymphoma [10]. Recent research also indicated that the administration of PD-1 blockades acts as a neoadjuvant to enhance the antitumor immune response and may represent a more efficacious approach to treating GBM [11]. However, most patients do not demonstrate a long-term response, and some become resistant to ICIs. One possible mechanism to explain this result is the consistent expression of PD-L1 through negative feedback regulation after the PD-1/PD-L1 axis blockade treatment [12]. Recent studies have suggested that PD-L1 is stabilized by glycosylation [13], CMTM4/6 [14, 15], and ZDHHC3 [16], which can be targeted to enhance the therapeutic effect of PD-1 blockade in tumors. However, it is still unknown whether the regulation of PD-L1 plays a role in ICI therapy for glioblastoma.

N6-methyladenosine (m6A) is the most prevalent, abundant, and conserved internal RNA modification in mammals [17]. The m6A modification, which is abundant in 5′UTRs, and 3′UTRs, and stops codons filed [18], can biologically function in RNA transcription [19], subcellular location [20], alternative splicing [21], stability [22], and translation efficiency [23]. The m6A modification involves tumorigenesis, progression, and chemoresistance by modifying the intrinsic mRNA or non-coding RNA [24]. Recently, increasing evidence has proven that perturbed m6A metabolism may participate in the immune response in some malignant tumors [25, 26]. Additionally, it was reported that ALKBH5 was upregulated in GBM, and higher ALKBH5 expression predicted poorer prognosis [27]. However, whether ALKBH5 influences the immune response in GBM remains largely understudied.

Herein, we confirmed that the deficiency of ALKBH5 sensitized immune response by elevating the population of infiltrated T lymphocytes. The underlying mechanisms were explored, and data suggested that knocking out ALKBH5 decreased ZDHHC3 mRNA expression in a YTHDF2-dependent manner, and ZDHHC3 augmented PD-L1 expression through diminishing PD-L1 degradation. Furthermore, we found that deletion of ALKBH5 enhanced the anti-PD-1 therapeutic effect, and the combination of anti-PD-1 antibody and IXO1, an ALKBH5 inhibitor, increased the survival benefits of GBM-bearing mice. Our results revealed a novel role of ALKBH5 in GBM and provided a promising combination strategy of immune therapy in GBM.

In the present study, we elucidate the functional significance of ALKBH5 in immune responses via the regulation of PD-L1 in glioma.

## Results

### ALKBH5 depletion suppresses tumor growth in immune-complete GBM-bearing mice

**Fig. 1:**
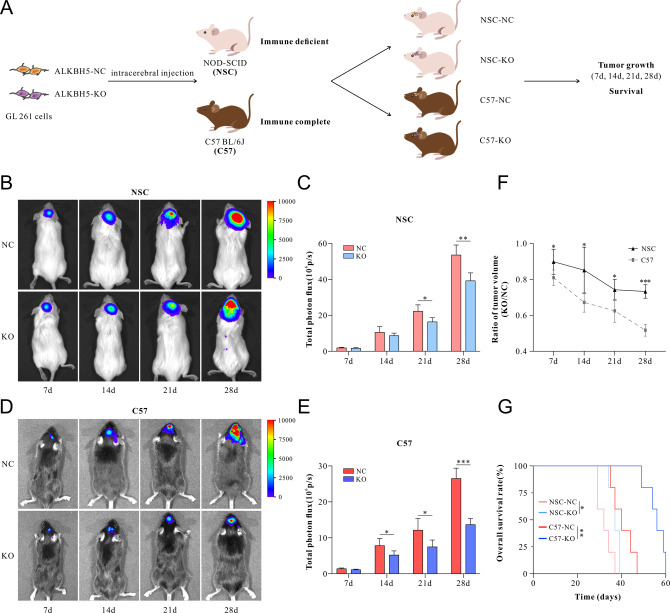
The role of ALKBH5 in immune-deficient and immune-complete GBM-bearing mice. **A** Experimental design to investigate the role of ALKBH5 in immune-deficient (NOD-SCID, NSC) and immune-complete (C57BL/6J, C57) tumor-bearing mice. **B**, **D** Representative pseudocolored bioluminescent images of the NSC (**B**) and C57 (**D**) mice after being implanted with ALKBH5-NC or ALKBH5-KO GL261 cells on days 7, 14, 21, and 28. **C**, **E** Quantitative analysis of bioluminescent signal intensity. **F** The photon flux signal intensity ratio in ALKBH5-KO and ALKBH5-NC treated group in NSC and C57 mice. **G** Kaplan–Meier overall survival curves of NSC and C57 mice after implanted with ALKBH5-NC or ALKBH5-KO GL261 cells. Data are presented as the mean ± SD. *n* = 8 per group. *p < 0.05, **p < 0.01, ***p < 0.001.

To determine the role of ALKBH5 in regulating immune response, we employed two orthotopic mouse models using GL261 murine glioma cells. After knocking out of ALKBH5 by CRISPR/Cas9 editing (Supplementary Fig. 1A, B), GL261 cells were intracranially inoculated into immune-deficient NOD-SCID (NSC) or immune-complete C57BL/J6 mice (C57) mice following the standard protocol (Fig. 1A). Tumor growths were calculated by bioluminescence imaging, and the survival time of tumor-bearing mice was recorded.

Compared with the ALKBH5-NC group, ALKBH5-KO (Knockout) tumor growth was significantly inhibited in NSC mice (Fig. 1B, C) on days 21 and 28. Similarly, ALKBH5-KO-treated tumors in C57 mice grew slower than in the ALKBH5-NC group (Fig. 1D, E). However, ALKBH5 knockout (KO) showed more dramatic effects in immune-complete (C57) mice than in immune-deficient (NSC) mice in suppressing tumor growth with a lower ratio of tumor volume (KO/NC) (Fig. 1F). Furthermore, the survival of ALKBH5-KO tumor-bearing mice was significantly prolonged when compared with the ALKBH5-NC group in NSC (37 vs. 32 days, *P* = 0.0273) and C57 mice (56 vs. 40 days, *P* = 0.0039) (Fig. 1G). Importantly, ALKBH5 deficiency showed more dramatic effects in prolonging survival time in immune-complete mice when compared with that in immune-deficient mice (Fig. 1G).

To determine whether ALKBH5 was associated with the advanced progression of glioma, the expression of ALKBH5 in different grades of glioma was analyzed by immunohistochemistry staining. As shown in Supplementary Fig. 1C, high ALKBH5 expression was associated with higher WHO grades. Recurrent glioma patients showed a relatively higher ALKBH5 expression than primary glioma patients (Supplementary Fig. 1D). Furthermore, Kaplan–Meier survival analysis revealed that the higher expression of ALKBH5 was correlated with poorer overall survival, as per TCGA data (Supplementary Fig. 1E), which was further validated by the CGGA dataset (Supplementary Fig. 1F, G).

### ALKBH5 deficiency inhibits tumor growth by rescuing antitumor immune response

Given that ALKBH5-deficient GBM demonstrated a sharper rate reduction in tumor growth and better survivability in C57 than in NSC mice, we hypothesized that ALKHB5 would influence the antitumor function of the immune system. As T cells play a vital role in the antitumor immune response, we performed immunohistochemistry assays (IHC) of mice GBM tissues.

**Fig. 2:**
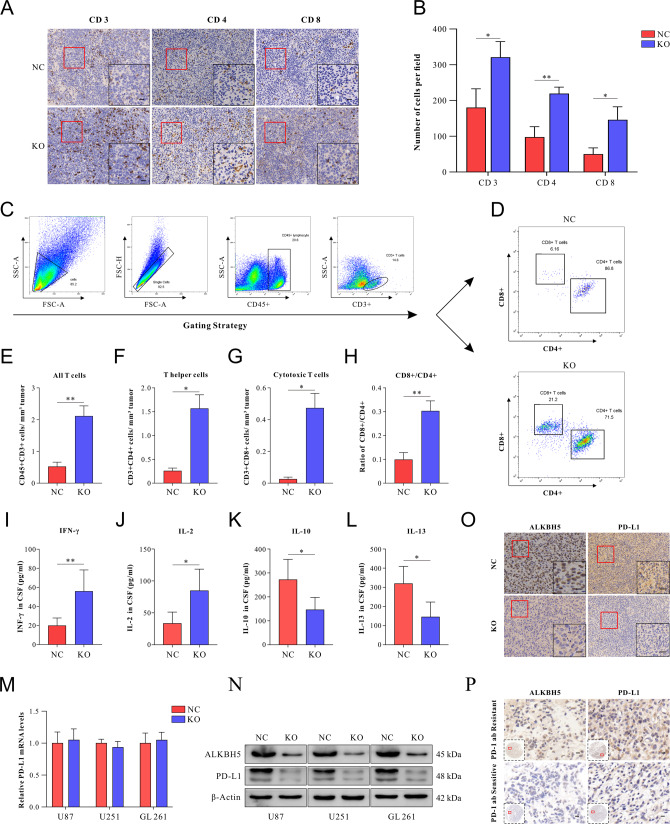
The deletion of ALKBH5 enhanced the recruitment and infiltration of T cells and was associated with decreased PD-L1 protein levels. **A** Representative immunohistochemical staining images of CD3^+^, CD4^+^, and CD8^+^ T cells in the tumor after transplantation of ALKBH5-NC and ALKBH5-KO GL261 cells on day 28. **B** Quantitative analysis of the numbers of positive cells per field in immunohistochemical staining images as shown in **A**. **C** Gating strategies of flow cytometric to analyze tumor-infiltrating CD45^+^CD3^+^CD4^+^CD8^−^ (CD4^+^) T helper cells and CD45^+^CD3^+^CD4^−^CD8^+^(CD8^+^) cytotoxic T cells in GBM tissues isolated from mice. **D** Representative flow cytometric image of tumor-infiltrating CD4^+^ and CD8^+^ T cells. FACS quantitative analysis of immune cells, including **E** CD3^+^ T cells, **F** CD4^+^ T cells, **G** CD8^+^ T cells, and **H** the ratio of CD8^+^/CD4^+^ T cells. The concentration of **I** IFN-γ, **J** IL-2, **K** IL-10, and **L** IL-13 in CSF of ALKBH5-NC and ALKBH5-KO GBM-bearing mice on day 28 were evaluated by ELISA. **M** The expression of PD-L1 mRNA in ALKBH-KO and ALKBH-NC GBM cells was measured by qRT-PCR. **N** PD-L1 protein levels were decreased after ALKBH5 knocking out in U87, U251, and GL261 cells. **O** Representative immunohistochemical staining images of ALKBH5 and PD-L1 in ALKBH5-KO and ALKBH5-NC GBM tissues harvested from mice. **P** Expression of ALKBH5 and PD-L1 in GBM tissues of anti-PD-1 Ab-sensitive and resistant patients. Scale bar = 60 μm. Data are presented as the mean ± SD. *p < 0.05, **p < 0.01.

IHC staining results indicated that the deletion of ALKBH5 significantly increased the number of tumor-infiltrating CD3^+^, CD4^+^, and CD8^+^ T cells (Fig. 2A, B) but not in the FOXP3^+^ cells (Supplementary Fig. 1H). Consistent with IHC results, flow cytometry of the GBM tissue indicates that knocking out ALKBH5 remarkably increased the number of infiltration T cells (Fig. 2C, D), including total T cells (CD45^+^CD3^+^) (Fig. 2E), T helper cells (CD45^+^CD3^+^CD4^+^CD8^−^) (Fig. 2F), and cytotoxic T cells (CD45^+^CD3^+^CD4^−^CD8^+^) (Fig. 2G). Importantly, the deletion of ALKBH5 increased the ratio of cytotoxic T cells (CD4^−^CD8^+^)/T helper cells (CD4^−^CD8^+^) (0.252 vs 0.066) (Fig. 2H). Additionally, proinflammatory and immune inhibitory cytokines in cerebrospinal fluid (CSF) were evaluated by enzyme-linked immunosorbent assay (ELISA) at 28 d after inoculation. The results showed that ALKBH5 deficiency significantly increased antitumor immune function cytokines, IFN-γ, and IL-2 levels (Fig. 2I, J). Instead, IL-10 and IL-13, associated with suppressed antitumor immune function, demonstrated a statistically significant decrease (Fig. 2K, L).

Recent studies have reported that ALKBH5 suppressed antitumor T cells by inhibiting PD-L1 mRNA degradation in N6-methyladenosine modification in intrahepatic cholangiocarcinoma [28]. Thus, PD-L1 expression in GBM cells and orthotopic mouse brain tumors was determined by Western blot and IHC staining. No significant changes in PD-L1 mRNA expression (Fig. 2M), while the protein level of PD-L1 demonstrated a remarkable reduction in ALKBH5-KO treated group compared with the ALKBH5-NC group (Fig. 2N). Consistently, IHC staining showed that PD-L1 expression was suppressed in ALKBH5-KO mice (Fig. 2O), which indicated that ALKBH5 might regulate PD-L1 expression post-translationally in glioma. Furthermore, we applied tissue microarray of GBM patients to explore the relationship between ALKBH5 and PD-L1. The results indicated that ALKBH5 was positively correlated with PD-L1 expression (Fig. 2P). Importantly, PD-1Ab-resistant GBM patients showed higher expression of ALKBH5 and PD-L1 when compared to PD-1 Ab-sensitive patients (Fig. 2P).

In conclusion, ALKBH5 deficiency improved the immune microenvironment by increasing the number of CD4^+^ and CD8^+^ T lymphocytes and increasing the ratio of CD8^+^/CD4^+^ T cells. We also found that knocking out ALKBH5 activates the immune system with more antitumor-associated cytokines (IFN-γ, IL-2) and fewer pro-tumor-associated cytokines (IL-10, IL-13). Besides, ALKBH5 deletion reduced PD-L1 protein levels but not mRNA expression in vitro and in vivo. Clinically, the positive relationship between ALKBH5 and PD-L1 was confirmed, and high expression levels of ALKBH5 in patients may be a predictor of PD-1 Ab resistance.

### Deletion of ALKBH5 enhances the efficacy of anti-PD-1 therapy in vivo

**Fig. 3:**
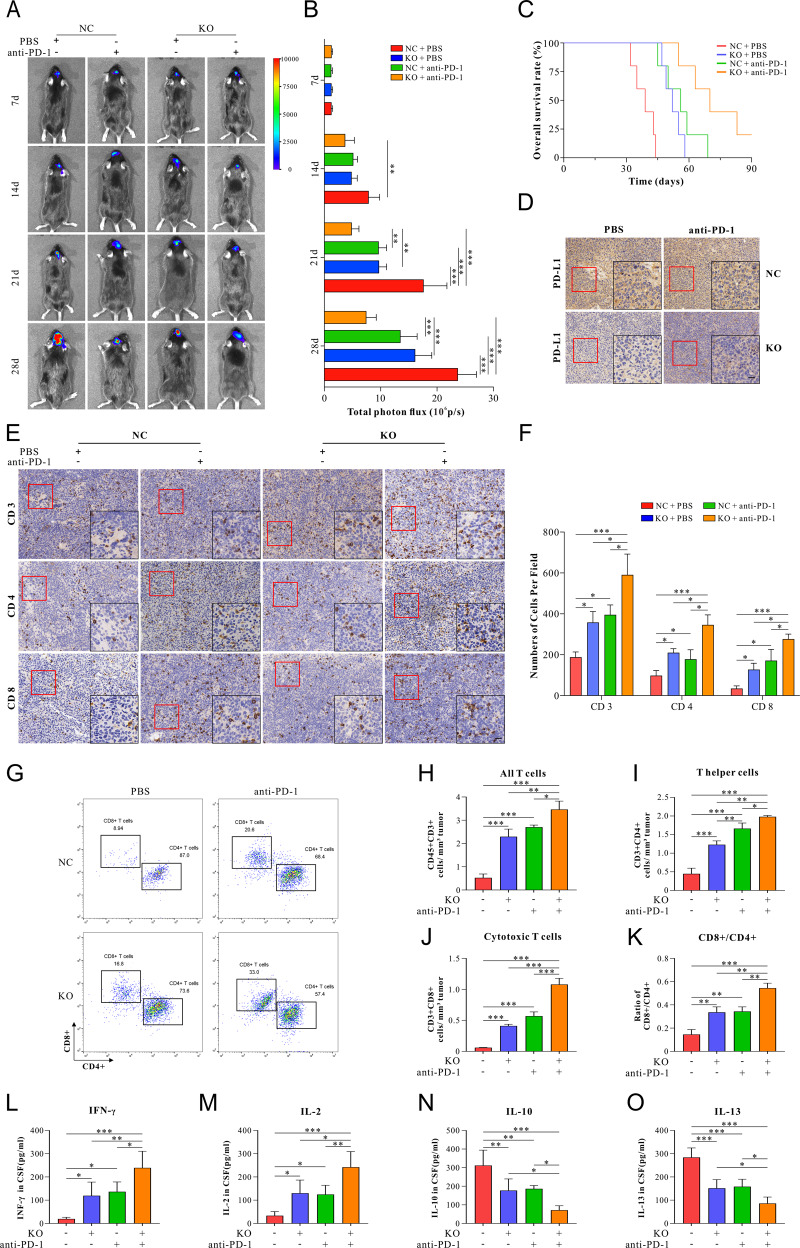
Knocking out ALKBH5 enhanced the effect of anti-PD-1-therapy in the GBM mice model. **A** Representative images of ALKBH5-NC or ALKBH5-KO GBM-bearing C57 mice with or without anti-PD-1 treatment on 7, 14, 21, and 28 d. GBM-bearing mice were treated with anti-PD-1 Ab on days 10, 15, and 20 at a 5 mg/kg dose. **B** Quantification of tumor-associated bioluminescence radiance intensity. **C** The Kaplan–Meier curve analyzed overall survival in C57 mice after being implanted with ALKBH5-NC and ALKBH5-KO GL261 cells with or without anti-PD-1 treatment. **D** Expression of PD-L1 was measured by immunohistochemical staining in GBM tissues harvested on day 28 from C57 mice implanted with ALKBH5-NC or ALKBH5-KO GL261 cells with or without anti-PD-1 treatment. **E** Representative immunohistochemical staining images of tumor-infiltrating CD3 T cells, CD4 T cells, and CD8 T cells in GBM tissues harvested on day 28 from ALKBH5-NC or ALKBH5-KO GBM-bearing mice with or without anti-PD-1 therapy. Scale bar = 60 μm. **F** Quantitative analysis of the numbers of positive cells per field in immunohistochemical staining images as shown in **E**. **G** Representative flow cytometric image of tumor-infiltrating CD4^+^ T cells and CD8^+^ T cells in ALKBH5-KO and ALKBH5-NC GBM tissues isolated from mice after anti-PD-1 therapy or PBS on day 28. **H**–**K** FACS quantification of tumor-infiltrating immune cells, including **H** CD3^+^ T cells, **I** CD4^+^ T cells, **J** CD8^+^ T cells, and **K** the ratio of CD8^+^ /CD4^+^ T cells isolated from ALKBH5-NC and ALKBH5-KO GBM tissues of mice after anti-PD-1 therapy or PBS on day 28. **L**–**O** The concentration of **L** IFN-γ, **M** IL-2, **N** IL-10, and **O** IL-13 evaluated by ELISA in CSF isolated from NC and ALKBH5-KO GBM-bearing mice after anti-PD-1 therapy or control on day 28. Data are presented as the mean ± SD. *n* = 8 per group. n = 8 per group. *p < 0.05, **p < 0.01, ***p < 0.001.

To determine the role of ALKBH5 in tumor cells in response to anti-PD-1 Ab treatment, We employed a mouse model using murine GBM cell line GL261. As described above, GL261 cells were knockout of ALKBH5 by CRISPR/Cas9 editing and orthotopically injected into immune-complete C57BL/6 (C57) mice. The mice were treated with anti-PD-1 Ab or PBS on days 10, 15, and 20. Compared with C57-GL261 tumors, the growth of ALKBH5-NC tumors was significantly reduced by anti-PD-1 Ab treatment (Fig. 3A, B), and the survival of tumor-bearing mice was very prolonged (Fig. 3C). Deleting ALKBH5 with anti-PD-1 Ab treatment further decreased the tumor growth rate and increased the survivability of tumor-bearing mice (Fig. 3A–C). In addition, PD-L1 expression was significantly reduced in ALKBH5-KO-treated mice compared with the ALKBH5-NC group. However, the treatment of anti-PD-1 Ab did not affect PD-L1 expression (Fig. 3D).

IHC staining showed that the anti-PD-1 Ab treatment could further increase the number of T lymphocytes (CD3^+^, CD4^+^, and CD8^+^ T cells) (Fig. 3E, F). Consistently, flow cytometric analysis of GBM tissues also showed that deletion of ALKBH5 with anti-PD-1 Ab treatment provided the most significant number of infiltration T cells (CD3^+^, CD4^+^, and CD8^+^ T cells) and notably increased the ratio of CD8^+^ /CD4^+^ T cells to the highest level among these four groups (Fig. 3G–K). ELISA showed a maximum increase of IFN-γ and IL-2 (Fig. 3L, M), while a maximum decrease of IL-10 and IL-13 in the group of ALKBH5-KO with anti-PD-1 treatment (Fig. 3N, O).

The above results confirmed that ALKBH5 deletion could enhance the therapeutic effect of anti-PD-1 therapy in tumor-bearing mice by reshaping the tumor immune microenvironment.

### ZDHHC3 is the potential target of ALKBH5 in glioma

**Fig. 4:**
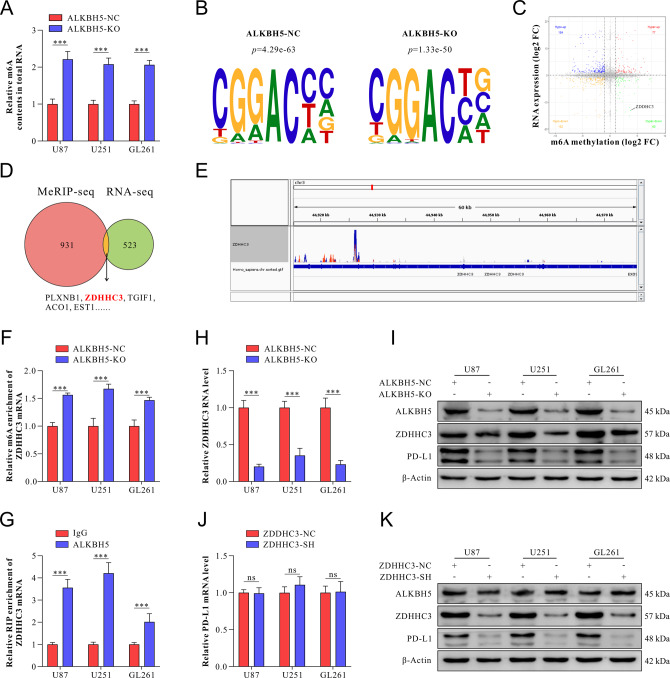
Transcriptome-sequencing and MeRIP-seq identified ZDHHC3 as a direct target of ALKBH5. **A** M6A quantification assays evaluated the relative enrichment of m6A methylation of total RNA in ALKBH5-NC and ALKBH5-KO U87, U251, and GL261 cell lines. **B** The predominant m6A consensus motif, “GAAC” was identified in U251 cells with or without ALKBH5 knockout. **C** The scatter diagram showed the distribution of genes with both differential m6A peaks (hyper or hypo, Y-axis, fold-change >1.5 or <2/3, p < 0.05) and differential expression (up or down, *X* axis, fold-change >2 or <0.5, *p* < 0.05) in ALKBH5-KO U251 cells compared with ALKBH5-NC U251 cells. **D** Overlap between m6A-upregulated genes and mRNA transcripts (fold-change>2 and *p* < 0.05). **E** The m6A abundance of ZDHHC3 mRNA with ALKBH5 knockout or control U251 cells was plotted by the IGV. The red and blue colors indicated the m6A signal of the ALKBH5-NC and ALKBH5-KO groups, respectively. **F** The enrichment of m6A modification of ZDHHC3 was measured by MeRIP-qPCR analysis, and **G** deletion of ALKBH5 increased m6A modification enrichment compared with controls in GBM cells. **H** Relative expression of ZDHHC3 mRNA was detected by qRT-PCR in ALKBH5-NC and ALKBH5-KO GBM cell lines. **I** ALKBH5, ZDHHC3, and PD-L1 protein levels was measured by western blot in ALKBH5-NC and ALKBH5-KO GBM cell lines. **J** Relative expression of PD-L1 mRNA was detected by qRT-PCR in NC and ZDHHC3-SH GBM cell lines. **K** ALKBH5, ZDHHC3, and PD-L1 protein levels were measured by western blot in ZDHHC3-NC and ZDHHC3-SH GBM cell lines. Data are presented as mean ± SD. *p < 0.05, **p < 0.01, ***p < 0.001.

As ALKBH5 increased PD-L1 protein levels but not mRNA levels, and the classic function of ALKBH5 was regulating targeted RNA by m6A modification, we hypothesized that there could be a potential direct target of ALKBH5, which could handle PD-L1 in a posttranslational manner. Thus, total RNA m6A levels were determined by m6A quantitative assay after ALKBH5 knockout. The results showed that the m6A levels of total RNAs increased in ALKBH5-KO treated cells compared with the ALKBH5-NC group (Fig. 4A).

To gain insight into the molecular mechanisms underlying the increased PD-L1 protein levels by m6A RNA demethylase ALKBH5, total RNA samples were isolated from ALKBH5-NC and ALKBH5-KO U251 cells for m6A profiling and transcriptome profiling using m6A-methylated RNA immunoprecipitation sequencing and RNA sequencing (RNA-Seq). Motif searching identified that the consensus “GGAC” motif was highly enriched within the m6A sites in ALKBH5-KO U251 cells (Fig. 4B).

Considering the demethylation role of ALKBH5 in m6A modification, we identified 77 hypermethylated genes whose mRNA transcripts were upregulated (*p* < 0.05, hyper-up) and 60 hypermethylated genes whose mRNA transcripts were downregulated (*p* < 0.05, hyper-down), which were considered as the potential targets (Fig. 4C). Among the top 10 candidate genes, we found that ZDHHC3 was markedly downregulated (Fig. 4D). Consistently, a high m6A peak was detected around 3’UTR of ZDHHC3 mRNA in ALKBH5-KO compared with ALKBH5-NC U251 cells (Fig. 4E). Moreover, relative m6A enrichment of ZDDHC3 mRNA was increased in the ALKBH5-KO group compared with ALKBH5-NC treated group in all three cell lines (Fig. 4F). Importantly, ZDHHC3, a palmitoyltransferase, which was recently reported, could palmitoylate PD-L1, thereby blocking the ubiquitination of PD-L1, consequently suppressing the PD-L1 degradation [16]. Interestingly, qRT-PCR and western blot results showed that the deletion of ALKBH5 suppressed ZDHHC3 expression in mRNA and protein levels (Fig. 4H, I). Moreover, RIP assays determined the binding of ALKBH5 with ZDHHC3 mRNA in GBM cell lines (Fig. 4G).

To investigate the role of ZDHHC3 in ALKBH5 regulation of PD-L1, specific shRNAs were designed to knockdown ZDHHC3, and the most knockdown efficiency was determined by qRT-PCR and western blot assay (Supplementary Fig. 2A, B). Then, the expression of PD-L1 and ALKBH5 was explored, and the results showed that knocking-down ZDHHC3 significantly decreased PD-L1 protein levels but with no significant changes in PD-L1 mRNA expression (Fig. 4J). Moreover, no significant differences in ALKBH5 protein levels after ZDHHC3 were knocked down when compared with the ZDHHC3-NC group (Fig. 4K).

Analysis of TCGA and CGGA databases suggested that elevated expression of ZDHHC3 positively correlated with glioma malignancy according to WHO grading and predicted poor prognosis of glioma patients (Supplementary Fig. 2D). IHC staining of the glioma surgical specimen also indicated that ZDHHC3 expression was higher in WHO III-IV glioma than in WHO II glioma and was higher in recurrent GBM than in primary GBM (Supplementary Fig. 2E, F).

Therefore, ZDHHC3 was selected as the candidate target of ALKBH5 in glioma for further investigation.

### ZDHHC3 knockdown suppresses PD-L1 expression by accelerating the degradation of PD-L1

As the results indicated that ZDHHC3 might be involved in ALKBH5 regulating PD-L1 protein levels, we hypothesized that ZDHHC3 might regulate PD-L1 in two ways: (1) ZDHHC3 may affect PD-L1 mRNA translation efficiency; (2) ZDHHC3 may influence PD-L1 protein stability.

**Fig. 5:**
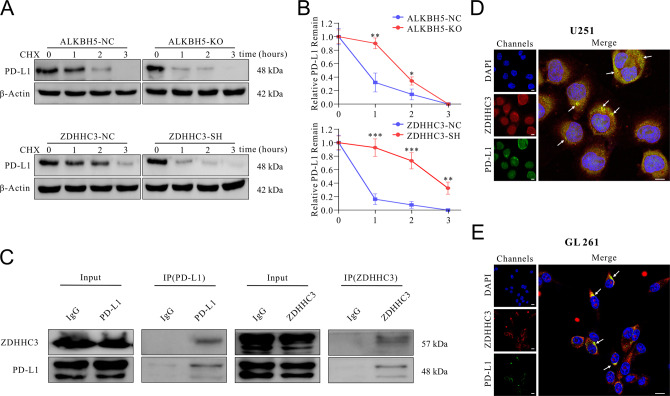
Knocking-down ZDHHC3 suppressed PD-L1 expression by promoting the degradation of PD-L1. **A** U251 cells with ALKBH5 knockout or control (top panel) and U251 cells with ZDHHC3 knockdown or control (bottom panel) were incubated with cycloheximide (CHX), and protein was extracted at the indicated time points. After that, PD-L1 protein expression was analyzed by western blot. This experiment was repeated three times. **B** Quantification of the western blot results in **A** as mean ± SD. **C** A co-immunoprecipitation assay using ZDHHC3 or PD-L1 as bait protein showed the reciprocal physical interaction between ZDHHC3 and PD-L1 in U251 cells. Immunofluorescence of ZDHHC3 and PD-L1 in U251 (**D**) and GL261 (**E**) cells. Scale bar = 10 μm. White arrows showed co-localization between ZDHHC3 and PD-L1.

To clarify whether ZDHHC3 regulates the protein stability of PD-L1 in glioma, cycloheximide-chase assays were conducted, and the results demonstrated that ALKBH5-KO and ZDHHC3-SH treatment accelerated the degradation of the PD-L1 when compared with their control groups (Fig. 5A, B). Based on these results, we assumed that the palmitoyltransferase ZDHHC3, which palmitoylates protein and regulates the stability of PD-L1, but not ALKBH5, whose primary function is m6A modification of RNA. Moreover, the co-immunoprecipitation (Co-IP) assay confirmed the physical interactions between ZDHHC3 and PD-L1 (Fig. 5C), and immunofluorescence indicated the co-localization of ZDHHC3 and PD-L1 in GBM cell lines (Fig. 5D, E).

These results consistently indicated that knocking-down ZDHHC3 reduced PD-L1 protein levels by accelerating its degradation.

### ALKBH5 deficiency facilitates ZDHHC3 mRNA degradation in a YTHDF2-dependent manner

**Fig. 6:**
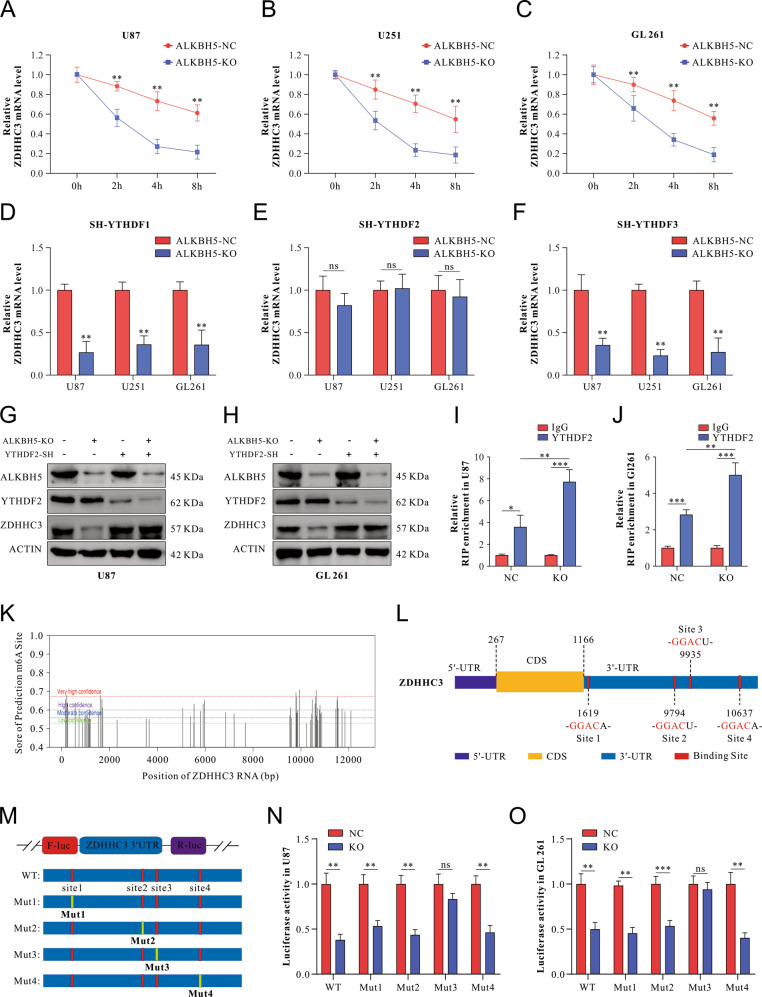
ALKBH5 deficiency facilitates ZDHHC3 mRNA degradation in a YTHDF2-dependent manner. **A**–**C** U87 (**A**), U251 (**B**), and GL261 cells (**C**) with or without ALKBH5 knockout were incubated with Actinomycin D, and RNA was isolated at the indicated time points. Then ZDHHC3 mRNA expression was measured by qRT-PCR. **D**–**F** U87, U251, and GL261 cells were co-transfected with ALKBH5-KO and YTHDF1-SH (**D**), YTHDF2-SH (**E**), and YTHDF3-SH (**F**), respectively. ZDHHC3 mRNA was measured by qRT-PCR, and data are presented as mean ± SD. **G**, **H** U87 (**G**) and GL261 (**H**) cells were co-transfected with ALKBH5-KO and YTHDF2-SH, ALKBH5, YTHDF2, and ZDHHC3 protein were evaluated by western blot. **I**, **J** RIP-qPCR was utilized to verify the ZDHHC3 mRNA enrichment by YTHDF2 with or without ALKBH5-KO in U87 (**I**) and GL261 (**J**) cell lines. The data were analyzed using a t-test and shown as mean ± SD. **K** The potential m6A sites of ZDHHC3 are predicted by the SRAMP analysis tool. **L** Schematic representation of selected high prediction score sites of m6A motifs within ZDHHC3 mRNA. **M** Schematic representation of mutated (GGAC to GGCC) m6A sites within 3′UTR of pmirGLO vector to investigate the roles of m6A in 3′UTR in ZDHHC3 expression. **N**, **O** The relative luciferase activity of F-Luc compared to R-Luc of pmirGLO-3′UTR-WT, or pmirGLO-3′UTR-Mut-1/-2/-3/-4 in NC and ALKHBH5-KO U87 (**N**) and GL261 (**O**) cell lines. The data were shown as mean ± SD. **p < 0.01, ***p < 0.001.

As ALKBH5 deficiency suppresses ZDHHC3 mRNA expression with increased m6A modification content, and the crucial function of m6A methylation was regulation of mRNA stability, we hypothesized that ZDHHC3 mRNA might degrade with the demethylating change of ALKBH5, which was further confirmed (Fig. 6A–C).

Next, we tested potential m6A binding proteins, known as m6A readers, by constructing knocking-down plasmids. Consistent with our hypothesis, knocking-down YTHDF2 offsets the downregulation of ZDHHC3 mRNA in ALKBH5-KO cells (Fig. 6E), whereas no significant changes in ZDHHC3 mRNA after knocking-down YTHDF1 and YTHDF3 (Fig. 6D, F). Western blot results also supported the role of YTHDF2 as a reader protein of ZDHHC3 (Fig. 6G). Furthermore, the binding of YTHDF2 and ZDHHC3 was identified by RNA-binding protein immunoprecipitation assays (Fig. 6I, J). These results demonstrated that YTHDF2 physically interacted with ZDHHC3 mRNA GBM cells.

An online m6A site prediction tool SRAMP (http://www.cuilab.cn/sramp/), was used to predict the potential m6A binding site and four binding sites located in 3’UTR of ZDHHC3 (consistent with our results), and the highest prediction scores were selected (Fig. 6K). Synonymous mutations (adenine replaced with thymine) were introduced at four selected m6A sites. The ZDHHC3 3’UTR with different mutations or wild type was inserted into pmirGlo dual-luciferase reporter, and the luciferase activity was measured in ALKBH5-KO and ALKBH5-NC GBM cells (Fig. 6L). Compared with the ALKBH5-NC treated cells, ALKBH5-KO facilitated a decrease of luciferase activity in the wild type, Mut1, Mut2, and Mut4 3’UTR in U87 and GL261 cells (Fig. 6M, N). Only Mut3 3’UTR abrogated the drop of luciferase activity mediated by ALKBH5 in both U87 and GL261 cells (Fig. 6N, O), which suggests that site 3 (GGACU, position at 9935 of ZDHHC3) in ZDHHC3 3’UTR is indispensable for ALKBH5-mediated m6A modification.

### ALKBH5 inhibitor, IOX1, improves the efficacy of anti-PD-1 Ab in the GBM mice model

**Fig. 7:**
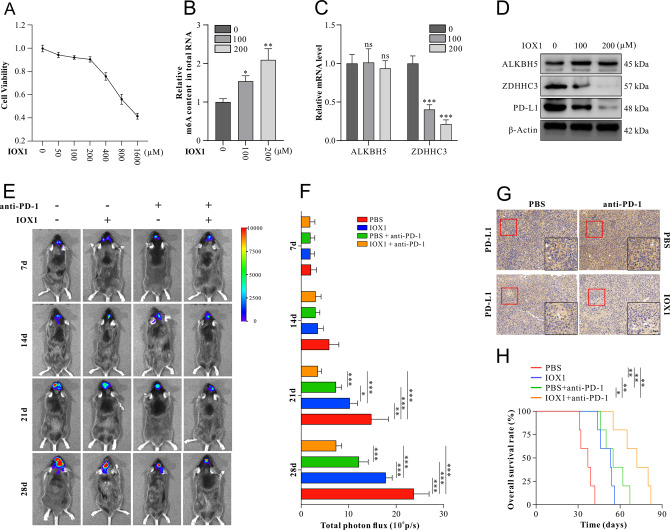
An ALKBH5 inhibitor IOX1 improves the efficacy of anti-PD-1 therapy in GBM mice model. **A** Proliferation assays of U87 cells treated with DMSO control, 50 μM, 100 μM, 200 μM, 400 μM, 800 μM, or 1600 μM IOX1. **B** M6A quantification assays evaluated the relative enrichment of m6A methylation of total RNA in U87 cells treated with DMSO control, 100 μM, and 200 μM IOX1, respectively. **C** Relative expression of ALKBH5 and ZDHHC3 mRNA was measured by qRT-PCR in U87 cells treated with DMSO control, 100 μM, and 200 μM IOX1, respectively. **D** ALKBH5, ZDHHC3, and PD-L1 protein expression were detected by western blot in U87 cells treated with DMSO control, 100 μM, and 200 μM IOX1, respectively. **E** Representative pseudocolor bioluminescence images of GBM-bearing C57 mice on days 7, 14, 21, and 28 after GL261 cells were implanted (day 0). GBM-bearing mice have injected with anti-PD-1 Ab (at a dose of 5 mg/kg) on day 10, day 15, and day 20 with or without intraperitoneal injection of IOX1 (at a dose of 10 mg per kilogram of mice weight) on day 5, day 7, and day 9. **F** Quantification of tumor-associated bioluminescence radiance intensity with data presented as mean ± SD. **G** PD-L1 expression was measured by immunohistochemical staining in GBM tissues isolated from mice treated with IOX1 and anti-PD-1 therapy. **H** The Kaplan–Meier curve analyzed overall survival in GBM-bearing mice treated with IOX1 and/or anti-PD-1. Scale bar = 60μm. Data are presented as mean ± SD. *n* = 8 per group. *p < 0.05, **p < 0.01, ***p < 0.001.

To date, our data has confirmed that the deletion of ALKBH5 in GBM cell lines enhances the efficacy of anti-PD-1 therapy in GBM-bearing mice. To explore the potential clinical applications, we selected IOX1, a specific ALKBH5 inhibitor, which inhibited ALKBH5 activity in a cofactor 2-oxoglutarate oxygenase competitive manner. The cytotoxicity of IOX1 in vitro was explored by CCK-8 assay, and the proliferation was not significantly inhibited at a concentration below 400 μM (Fig. 7A). IOX1, at a concentration of 100 μM, significantly increased total RNA m6A content (Fig. 7B) and reduced ZDHHC3 expression in mRNA and protein levels, decreasing PD-L1 protein, whereas IOX1 did not affect ALKBH5 mRNA and protein expression levels (Fig. 7C, D). Increasing the concentration of IOX1 to 200 μM strengthened these effects.

Next, we compared tumor growth of IOX1-treated mice and PBS control with or without anti-PD-1 Ab immunotherapy. Bioluminescence and Kaplan–Meier survival analysis indicated that IOX1 slowed GBM growth, prolonged survival time, and enhanced these effects of anti-PD-1 therapy (Fig. 7E, F, H). Importantly, IHC staining showed that IOX1 significantly decreased the expression of PD-L1 with the treatment of anti-PD-1 compared with the anti-PD-1 + PBS group (Fig. 7G).

These results suggest that the ALKBH5 inhibitor, IOX1, could significantly reduce PD-L1 expression via ZDHHC3, slow tumor growth rate, and prolong mice survival time. More importantly, we provide a rational strategy of combination therapy using anti-PD-1 Ab and ALKBH5 inhibitor, IOX1.

## Discussion

In the present study, we showed that the knockout of m6A demethylase ALKBH5 dramatically inhibited tumor growth in immune-complete mice with increased infiltration of T cells in xenograft. ALKBH5 deficiency decreased PD-L1 protein level by suppressing ZDHHC3 mRNA expression in an m6A modification manner, further remodeling the tumor immune microenvironment. Finally, ALKBH5 improved the tumor immune microenvironment and enhanced the efficacy of anti-PD-1 therapy through the acceleration of PD-L1 degradation, which provides a promising combination therapeutic strategy for GBM.

A pivotal role in stabilizing PD-L1 protein is regulating ZDHHC3 expression levels in m6A modification machinery. Studies have concluded that substantially expressed protein and unexpected stabilization of PD-L1 contribute to the immunosuppressive microenvironment of the GBM [29–31]. Our results suggested that targeting ALKBH5 improved the tumor immune microenvironment and enhanced the efficacy of anti-PD-1 therapy through the acceleration of PD-L1 degradation, which provides a promising combination therapeutic strategy for GBM.

ALKBH5, as RNA demethylase, was found high-expressed in various types of cancers and considered a negative prognosis biomarker. However, most studies focused on the function of ALKBH5 within tumor cells, not the tumor microenvironment. Recently, a study reported that ALKBH5 could promote a tumor-suppressive immune microenvironment by transporting lactate into extracellular space in melanoma [25]. Furthermore, ALKBH5 was identified to directly regulate the mRNA of PD-L1, a critical immune checkpoint molecule, and thus influence the immune microenvironment and immunotherapy in the intrahepatic cholangiocarcinoma [28]. In glioma, it was found that ALKBH5 remodeled the immune microenvironment via paraspeckle assembly and IL8 secretion yield [32]. Thus, various stress-induced epigenetic modifications, including m6A modification by ALKBH5, may be the reason for cancer’s immunotherapy resistance, which still requires further investigation.

The PD-1/PD-L1 axis is a promising target for tumor immunotherapy and has shown an unprecedented antitumor response rate in advanced cancers. However, many of these cancer patients cannot get a durable response to such immunotherapies. Several studies have found that intrinsic regulation of PD-L1 is an essential factor contributing to the resistance of anti-PD-1 therapy. CMTM4, CMTM6, and ZDHHC3 were identified as intrinsic regulators that maintain the expression of PD-L1 and as promising therapeutic targets for immunotherapy. Interestingly, ZDHHC3 inhibition not only accelerates the degradation of PD-L1 on the cell membrane but depletes its storage in recycling endosomes, which are responsible for the resistance of the immunotherapy field [14–16]. Our results built a link between ALKBH5 and ZDHHC3 and uncovered a novel function of ALKBH5 as an immune environment modulator.

ALKBH5 is characterized as RNA demethylase, and ALKBH5-mediated RNA m6A demethylation affects the binding of m6A reader protein. Studies believe that the level of m6A modification that stabilizes RNA or accelerates RNA decay depends on the type of m6A reader proteins [17, 33]. The m6A reader, YTHDF2, reportedly interacts with the RNA site with high m6A modification, and the binding of YTHDF2 promotes the decay of RNA [34]. Our results confirmed that ALKBH5-mediated m6A demethylation stabilizes ZDHHC3 mRNA by reducing the binding between ZDHHC3 mRNA and YTHDF2, followed by an increase in ZDHHC3 mRNA level. In addition, our data verified that both ALKBH5 and YTHDF2 were directly bound to ZDHHC3 mRNA and uncovered a specific ALKBH5 binding site in ZDHHC3 3’UTR. It remains to be determined whether the alteration of m6A modification influenced ZDHHC3 mRNA levels through regulating alternative splicing and translation efficiency.

One study held by [25] reported that ALKBH5 negatively influences the efficacy of anti-PD-1 therapy in melanoma by regulating the tumor-infiltration regulatory T cells (Tregs) and Myeloid-derived suppressor cells mediated by lactate metabolism. This study does not observe a change in the infiltration of cytotoxic CD8+ T cells. A possible interpretation may locate in the difference in the tumor microenvironment. As is well known, brain tumors, including GBM, are in a unique brain environment with immune privilege, categorized as “cold tumors,” whereas melanoma is a typical “hot tumor”. Thus, the change of infiltration of CD8 + cytotoxic T cells may not be evident in a tumor microenvironment with abundant T lymphocytes. The limitations of the present study were that we did not explore whether lactate metabolism participates in the regulation of ALKBH5 in the GBM immune microenvironment, which needs further exploration.

Additionally, the deletion of METTL3 or METTL14 reportedly promoted IFN-γ signaling by stabilizing the stat1 and IRFL mRNA by YTHDF2 [35]. Conversely, we found that deletion of ALKBH5, which is a demethylase but not a methylase, increased IFN-γ levels in CSF. However, this mechanism was not explored in our study, and it remains to be elucidated whether the alteration of cytokines is derived from the increased number of infiltration T cells or the intrinsic regulation by ALKBH5.

In our study, knocking out ALKBH5 did not affect tumor growth in immune-deficient NSC mice, emphasizing the pivotal role of m6A modification in regulating the antitumor immune response. However, previous research on ALKBH5 in GBM reported that the deletion of ALKBH5 disrupted GBM stem-like cell tumorigenicity and inhibited tumor progression [27]. ALKBH5-mediated promotion of tumor proliferation was also reported in hepatocellular carcinoma [36], pancreatic cancer [37], and other cancers [38, 39]. An important reason for these discrepancies was that we employed murine syngeneic GBM cells GL261 for the mice experiments but not human syngeneic GBM cell lines. However, various physiological tumor microenvironments and external stress may influence the function of m6A methylases or demethylases. Still, exploring the biological processes of ALKBH5 in tumorigenesis or tumor progression through intrinsic regulation of tumor cells or the remodeling of the tumor microenvironment is of great value.

In conclusion, our study revealed a new function of ALKBH5 in regulating PD-L1 degradation and improved the immune microenvironment via m6A-mediated alteration of ZDHHC3 mRNA stability in GBM. Additionally, ALKBH5 inhibition potentiated the response of anti-PD-1 therapy, suggesting that IOX1 could be a potential combination strategy with ICI therapy in GBM.

## Materials and methods

### Cell culture and transfection

Two human GBM cell lines, U87 and U251, and one mouse GBM cell line, GL261 (Cell Bank of Chinese Academy of Sciences, Shanghai, China), were used in this study. The cell lines U251 and GL261 were cultured in Dulbecco’s modified Eagle’s medium (DMEM, Gibco, Waltham, MA, USA). The U87 cell line was cultured in Eagle’s minimum essential medium (MEM, Gibco, Waltham, MA, USA), supplemented with 10% fetal bovine serum (FBS, Gibco, Waltham, MA, USA), penicillin (200 units/ml, Gibco, Waltham, MA, USA), and streptomycin (100 μg/ml, Gibco, Waltham, MA, USA) 5% CO_2_-humidified incubators (Thermo Scientific, Waltham, MA, USA) at 37 °C.

Lentiviral particles were designed and constructed by GeneChem (Shanghai, China). The sequences of sgRNAs, shRNAs, and siRNAs used for transfection were summarized in Supplementary Table 1. Before transfection, exponentially growing cells were seeded in a six-well cell culture cluster (NEST Biotech, Wuxi, China) at 50% confluence with a complete growth medium. According to the manufacturer’s instructions, the transfection complex was made of lentiviral activation vectors at optimal titer, 40 μl HiTransG P (transfection enhancer, GeneChem, Shanghai, China), and 960 μl Opti-MEM (Gibco, Waltham, MA, USA). At 12 h after transfection, the medium was replaced with DMEM/% FBS. The transfected cells were then screened with puromycin (2.00 μg/ml, #sc-108071, Santa Cruz, Dallas, TX, USA) for one week, and the transfection efficiency was confirmed by western blot and qRT-PCR.

### Mouse model and treatments

Animal studies and procedures were approved by the Animal Experimental Committee of Southern Medical University and complied with the Laboratory Animal Care and Use Guide. Female C57BL/6 J wild-type mice were purchased from Guangdong Medical Laboratory Animal Center (Guangzhou, China). NOD-SCID mice, which are T and B cells deficient, were obtained from The Jackson Laboratory. All mice were housed in a specific pathogen-free animal facility at the Experimental Animal Center of Zhujiang hospital. GL261 luciferase-expressing cells were transfected with lentiviral vectors (ALKBH5-KO, ZDHHC3-SH, and their Negative Control). All the mice were randomly and equally divided into different experimental groups. Approximately 5 × 10^5^ cells were injected into the right cerebrum of mice at day 0. Intracranial xenograft bioluminescence was performed weekly beginning on day 7. The mice were then injected intraperitoneally with 5 mg/kg (~100 μg/mouse) anti-human PD-1 monoclonal antibody (Ab) (Camrelizumab, Lianyungang, Jiangsu, China) on days 10, 15, and 20, or 10 mg/kg (~200 μg/mouse) ALKBH5 inhibitor IOX1 (MCE, NJ, USA) on days 5, 7, and 9. Each experimental group included eight mice. The survival time was recorded, and the mice’s brains were harvested for further investigations.

### Quantitative real-time PCR and RNA stability

Total RNA was isolated using TRIzol reagent (Takara Bio, Shiga, Japan), and cDNA was synthesized using Prime ScriptTM RT reagent kit (Takara Bio, Shiga, Japan) according to the manufacturer’s instructions. Quantitative real-time PCR (qRT-PCR) assay was performed using SYBR GREEN PCR Master Mix (Takara Bio, Shiga, Japan) on 7500 Fast Real-time PCR System (Applied Biosystem, Foster City, CA, USA). Glyceraldehyde-3-phosphate dehydrogenase (GAPDH) was used as an endogenous normalization control. The primers are listed in Supplementary Table 2. The cycle threshold (Ct) value was used for quantification through the 2^−△△Ct^ method, and the data were presented as the mean ± SD of at least three independent experiments. To explore the stability of RNA, actinomycin (5 μg/ml) was added to GBM cells. After incubation, total RNA was isolated from cells, and qRT-PCR was performed to evaluate stability.

### Protein extraction and western blot analysis

The proteins were extracted from cells using the Keygen total protein extraction kit (Keygen, Biotech, Jiangsu, China) and then quantified by a bicinchoninic acid protein assay kit (Thermo Scientific, Waltham, MA, USA) as previously described [40, 41]. The western blot experiments were performed according to standard procedures. The primary antibodies applied are shown in Supplementary Table 3. The membranes were incubated with HRP-labeled goat-anti-rabbit or goat-anti-mouse secondary antibodies (Cell Signaling Technology, Danvers, MA, USA). The proteins were detected and visualized using ImageQuant Las500 chemiluminescence (GE, Boston, USA). The protein expression was analyzed by the Image J software (National Institution of Health, USA), and β-actin was used as a control.

### Cytokine quantification by ELISA

Following the manufacturer’s instructions, the concentrations of IFN-γ, IL-2, IL-10, and IL-13 in cerebrospinal fluid were measured using ELISA kits (Signalway antibody, CA, USA) shown in Supplementary Table 4. A standard curve was calculated using recombinant cytokines of a known concentration. Based on the standard curve, the sample cytokine concentrations were evaluated.

### Dual-luciferase reporter assay

The wild-type or mutant 3’UTR of ZDHHC3 was inserted behind the F-Luc coding region using the priGLO system to evaluate the effect of 3’UTR on ZDHHC3 expression. Cells with ALKBH5 loss or wild type were co-transfected with these luciferase reporters. After incubation for 24 h, luciferase activity was measured by a dual-luciferase reporter gene assay kit (Promega, Madison, WI, USA), following the manufacturer’s instructions. Renilla luciferase activity was used as the control to normalize firefly luciferase activity.

### Co-IP assay

According to the manufacturer’s recommendations, the Co-IP assay was performed using the Dynabeads Antibody Coupling Kit (Thermo Fisher Scientific, Waltham, MA, USA). An appropriate volume of specific antibodies (Supplementary Table 3) was incubated with beads on a roller at 37 °C overnight. Cell lysates were incubated with antibody-coupled beads on a roller overnight at 4 °C to enrich the interacted protein complex. After washing, the beads were collected, and interacted protein complex was isolated from beads and analyzed by western blot.

### RIP assay

The RNA immunoprecipitation assay was performed using the EZ-Magna RIP RNA-binding protein immunoprecipitation kit (Millipore, MA, USA). After being coupled with antibodies, magnetic beads were incubated with cell lysates overnight at 4 °C. The RNA–protein complexes were isolated from the beads. After the digestion of protein in the complexes by proteinase K, RNA was extracted by TRIzol (Takara Bio, Shiga, Japan). The expression of ALKBH5 and target RNA was analyzed by qRT-PCR.

### Flow cytometry of tumor-infiltrating immune cells

Brain tumors were excised aseptically from mice, weighed, and mechanically diced. Single-cell suspensions were made using Brain Tumor Dissociation Kit (Miltenyi Biotech, Bergisch Gladbach, Germany) and gentleMACS^TM^ Octo Dissociator (Miltenyi Biotech, Bergisch Gladbach, Germany) according to the manufacturer’s instructions. The single-cell suspensions were filtered by a 70 μm filter and resuspended in FACS stain buffer. Red blood cells were lysed using an RBC removal solution. Cells were incubated with Fixable Viability Stain (BD Pharmingen, Franklin, NJ, USA). The cells were labeled with combinations of antibodies (Supplementary Table 3) against cell surface markers. At last, the cells were resuspended in FACS staining buffer and analyzed on the Beckman CytoFLEX (Brea, CA, USA). BD CompBeads were used to optimize fluorescence settings (BD Biosciences, Franklin, NJ, USA). Fluorescence-minus-one, unstained, and single-stained cells were also used to set gates.

### m6A quantification

The global m6A levels in total RNAs were measured using the EpiQuik m6A RNA Methylation Quantification Kit (Epigentek, NY, USA) following the manufacturer’s protocol. Total RNA was extracted by TRIzol (Takara Bio, Shiga, Japan) and treated with deoxyribonuclease I (Sigma, USA). Next, 200 ng RNAs were coated on each assay well. Each well was then filled with the capture antibody solution and detection antibody solution. After that, the m6A level was quantified by measuring the absorbance of each well at 450 nm according to the standard curve.

### IHC and immunofluorescence

According to standard procedures, freshly excised tumor tissues were fixed in paraformaldehyde, dehydrated, embedded in paraffin, sectioned into 5-μm slices, and mounted on slides. After, antigen retrieval was performed in citrate buffer for 3 min. Endogenous peroxidase activity and nonspecific antigens were blocked with 3% H_2_O_2_ and goat serum. The samples were incubated with the primary antibodies at 4 °C overnight. The samples were then washed and incubated with a biotinylated secondary antibody (1:500, Santa Cruz Biotechnology, USA) at room temperature for two h. Staining intensities were scored for statistical analysis.

Cells were cultured and fixed on confocal dishes, followed by overnight incubation with monoclonal antibodies (Supplementary Table 3) at 4 °C. Then, fluorophore-conjugated secondary antibodies were added to incubate for 30 min. Nuclear counterstaining was conducted with DAPI (Cell Signaling Technology, Danvers, MA, USA). Digital images were obtained using a fluorescence microscope (Zeiss LSM880, Oberkochen, Germany).

### Statistical analysis

All data were analyzed by SPSS 22.0 (SPSS, Chicago, IL, USA) and GraphPad Prism software 9.0 (GraphPad Software Inc., San Diego, CA, USA). Data are presented as the mean ± standard error unless otherwise indicated. Statistical significance was determined using the student’s two-tailed t-test for two groups and one-way ANOVA for multiple groups. Kaplan–Meier survival curves were used to evaluate the survival rate. All statistical tests were two-sided, and a p-value of <0.05 was considered statistically significant.

## Supplementary information

Supplemental Tables 1–4

Supplemental Figure S1

Supplemental Figure S2

Supplementary Figure Legends

Supplemental data for Uncropped Western Blots

## Data Availability

All data generated or analyzed during this study are included in this published article (and its supplementary information files).
