# Supplementary material for: ALKBH5 promotes PD-L1-mediated immune escape through m6A modification of ZDHHC3 in glioma
Source: Cell Death Discov. 2022 Dec 24;8:497. doi: 10.1038/s41420-022-01286-w (PMC9789960; doi:10.1038/s41420-022-01286-w)
Supplement: Supplementary file 1 — Supplemental Tables 1–4 [file 41420_2022_1286_MOESM1_ESM.docx]

**Supplemental Table 1. sgRNAs, shRNAs, and siRNAs used for transfection.**

| **ALKBH5** | |
| --- | --- |
| NC | CGCTTCCGCGGCCCGTTCAA |
| sg1 | CCACCGTGTGCTCGTTGTAC |
| sg2 | ACAACTATAAGGCGGGCAGC |
| sg3 | GGACACAGGGTAAGGTTCGG |
| **Zdhhc3** | |
| NC | TTCTCCGAACGTGTCACGT |
| sh1 | CCACAGTGATTCTCCTTAT |
| sh2 | CCTCAAAGTGGATTCACTT |
| **YTHDF1** | |
| NC | TTCTCCGAACGTGTCACGT |
| sh1 | GATACAGTTCATGACAATGA |
| sh2 | GAAACGTCCAGCCTAATTCT |
| **YTHDF2** | |
| NC | TTCTCCGAACGTGTCACGT |
| sh1 | GCTCTGGATATAGTAGCAATT |
| sh2 | CCTACTTACCCAGTTACTACA |
| **YTHDF3** | |
| NC | TTCTCCGAACGTGTCACGT |
| sh1 | CCTATGGACAAATGAGTAA |
| sh2 | GCAGTGGTATGACTAGCAT |

**Supplemental Table 2**. Primers used in real-time PCR for the target genes.

| **Gene Name** | **GenBank Number** | **Sense (5’-3’)** | **Anti-sense (5’-3’)** |
| --- | --- | --- | --- |
| **Homo sapiens** | | | |
| ALKBH5 | NM_017758.4 | TGAGCACAGTCACGCTTCCC | TCCGTGTCCTTCTTTAGCGACTC |
| ZDHHC3 | NM_001349377.2 | CCACTTCGCGAAACATTGAGCG | CCACAGCCGTCACGGATAAA |
| PD-L1 | NM_014143.4 | TGGCATTTGCTGAACGCATTT | TGCAGCCAGGTCTAATTGTTTT |
| YTHDF1 | NM_017798.4 | TGATCTAATGTGAAATGTAAG | CTTACATTTCACATTAGATCA |
| YTHDF2 | NM_001173128.2 | TAGCCAACTGCGACACATTC | CACGACCTTGACGTTCCTTT |
| YTHDF3 | NM_001277813.2 | ATCAGAGTAACAGCTATCCAC | CCCAGGTTGACTAAATACAC |
| GAPDH | NM_001289746.2 | GCATTGCCCTCAACGACCAC | CCACCACCCTGTTGCTGTAG |
| **Mus musculus** | | | |
| ALKBH5 | NM_172943.4 | GCGCGGTCATCAACGACTA | ATCAGCAGCATACCCACTGAG |
| ZDHHC3 | NM_001372547.1 | CGGGAGCCATGTGGTTTATCC | ACTCCGCATAGAGGACCAGAA |
| PD-L1 | NM_021893.3 | GCTCCAAAGGACTTGTACGTG | TGATCTGAAGGGCAGCATTTC |
| YTHDF1 | NM_173761.3 | ACAGTTACCCCTCGATGAGTG | GGTAGTGAGATACGGGATGGGA |
| YTHDF2 | NM_145393.4 | GAGCAGAGACCAAAAGGTCAAG | CTGTGGGCTCAAGTAAGGTTC |
| YTHDF3 | NM_001358041.1 | CATAGGGCAACAGAGGAAACAG | ATCTCCAGCCGTGGACCAT |
| GAPDH | NM_017008.4 | AGGTCGGTGTGAACGGATTTG | TGTAGACCATGTAGTTGAGGTCA |

**Supplemental Table 3**. Antibodies used in experiments.

| Antibodies | Manufacturer | catalog |
| --- | --- | --- |
| ALKBH5 | proteintech | 67811-1-Ig |
| ALKBH5 | milipore | HPA007196 |
| ZDHHC3 | santa cruz | sc-377378 |
| PDL1 | abcam | ab205921 |
| PDL1 | proteintech | 66248-1-Ig |
| YTHDF1 | abcam | ab220162 |
| YTHDF2 | abcam | ab220163 |
| YTHDF3 | abcam | ab220161 |
| ACTIN | CST | 3700S |
| CD3 | abcam | ab16669 |
| CD4 | abcam | ab183685 |
| CD8 | abcam | ab237709 |
| FOXP3 | abcam | ab215206 |
| Anti-N6-methyladenosine (m6A) | milipore | ABE572 |
| Fixable Viability stain 510 | BD horizon | 564406 |
| FITC Rat anti-mouse CD45 | BD pharmingen | 553079 |
| APC-Cy7 hamster anti-mouse CD3e | BD pharmingen | 557596 |
| APC Rat anti-mouse CD4 | BD pharmingen | 553051 |
| PerCp-Cy5.5 rat anti-mouse CD8a | BD pharmingen | 551162 |

**Supplemental Table 4**. ELISA kits used in cytokine measurements.

| **ELISA kits** | **Manufacturer** | **catalog** |
| --- | --- | --- |
| Mouse Interferon gamma ELISA Kit | Signalway antibody | EK1123 |
| Mouse Interleukin-2 ELISA Kit | Signalway antibody | EK1198 |
| Mouse Interleukin-10 ELISA Kit | Signalway antibody | EK1144 |
| Mouse Interleukin-13 ELISA Kit | Signalway antibody | EK1163 |
