# Supplementary material for: ALKBH5 promotes PD-L1-mediated immune escape through m6A modification of ZDHHC3 in glioma
Source: Cell Death Discov. 2022 Dec 24;8:497. doi: 10.1038/s41420-022-01286-w (PMC9789960; doi:10.1038/s41420-022-01286-w)
Supplement: Supplementary file 4 — Supplementary Figure Legends [file 41420_2022_1286_MOESM4_ESM.docx]

**Supplementary Figure 1** (A-B) The knockout efficiency of ALKBH5 using CRISPR/Cas9 was evaluated by qRT-PCR (A) and western blot assay (B). Representative immunohistochemical staining images of FOXP3 and Ly6G in the tumor after transplantation of ALKBH5-NC and ALKBH5-KO GL261 cells on day 28. (C-D) Representative immunohistochemical staining images of ALKBH5 in glioma patients (C), including primary and recurrent glioma patients (D). (E) Kaplan-Meier overall survival curves according to high and low ALKBH5 expression from the TCGA database. (F, G) Kaplan-Meier overall survival curves according to high and low ALKBH5 expression in primary glioma patients (F) or recurrent glioma patients (G) from the CCGA database. Scale bar = 60μm. Data are presented as mean ± SD. ^***^p < 0.001.

**Supplementary Figure 2** (A-B) The knockdown efficiency of ZDHHC3 using CRISPR/Cas9 was evaluated by qRT-PCR (A) and western blot assay (B). (C) Expression of ZDHHC3 in GBM (WHO IV grade glioma) and LGG (WHO II or III grade glioma) from TCGA and GTEx database. (D) Kaplan-Meier overall survival curves according to high and low ZDHHC3 expression from the TCGA database. (E-F) Representative immunohistochemical staining images of ZDHHC3 in glioma patients (E), including primary and recurrent glioma patients (F). Scale bar = 60μm. Data are presented as mean ± SD. ^***^p < 0.001.
