# Supplementary figures and images for: ALKBH5 promotes PD-L1-mediated immune escape through m6A modification of ZDHHC3 in glioma
Source: Cell Death Discov. 2022 Dec 24;8:497. doi: 10.1038/s41420-022-01286-w (PMC9789960; doi:10.1038/s41420-022-01286-w)

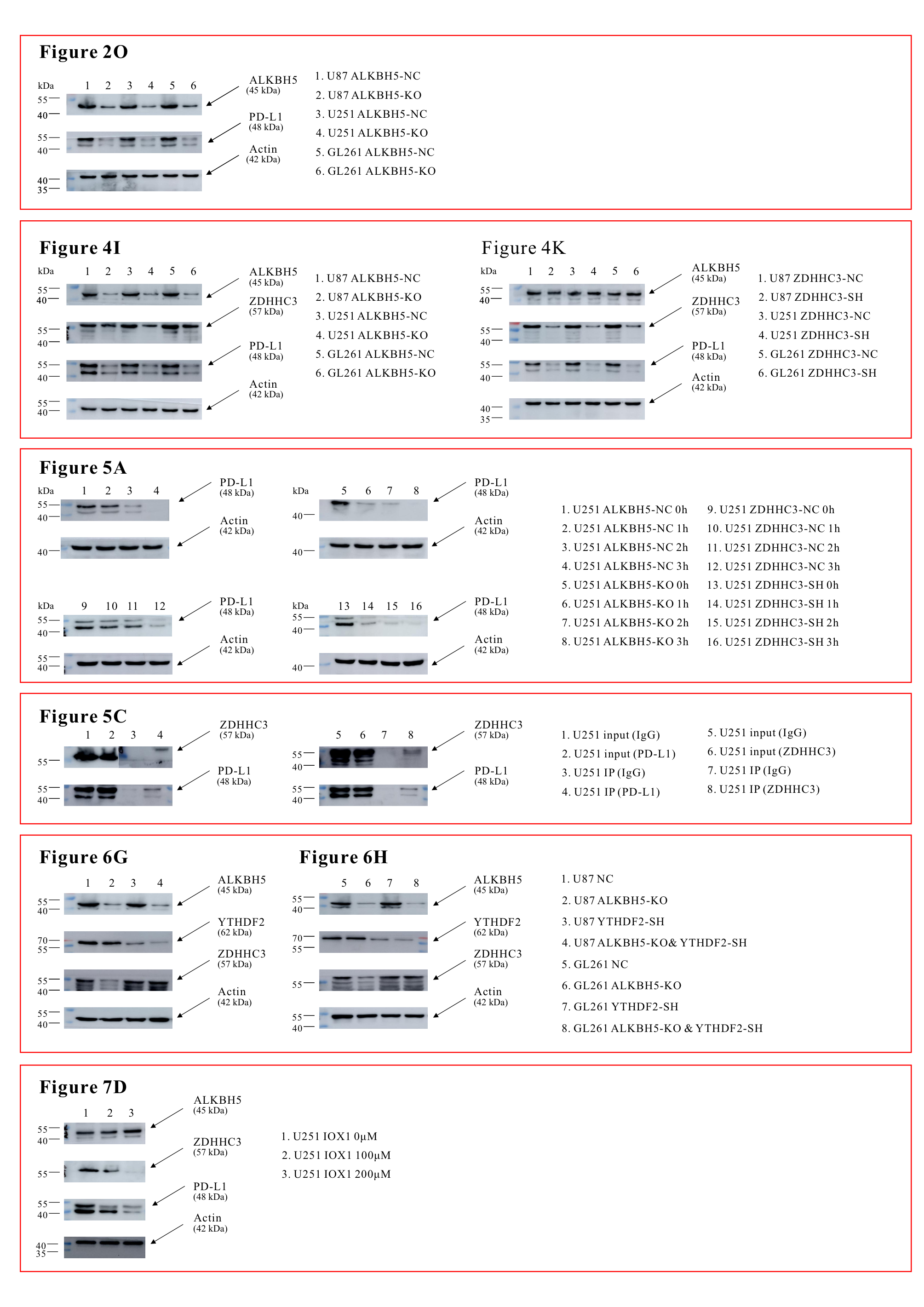

Supplement: Supplementary file 5 — Supplemental data for Uncropped Western Blots [file 41420_2022_1286_MOESM5_ESM.tif]
